# A Lightweight CP-ABE Scheme with Direct Attribute Revocation for Vehicular Ad Hoc Network

**DOI:** 10.3390/e25070979

**Published:** 2023-06-25

**Authors:** Yilong Liu, Shengwei Xu, Ziyan Yue

**Affiliations:** 1 School of Cyberspace Security, Beijing University of Posts and Telecommunications, Beijing 100876, China; lyl_160719@bupt.edu.cn (Y.L.); yzybjyd@gmail.com (Z.Y.); 2 Department of Cyberspace Security, Beijing Electronic Science and Technology Institute, Beijing 100070, China; 3 Institute of Information Security, Beijing Electronic Science and Technology Institute, Beijing 100070, China

**Keywords:** VANETs, CP-ABE, attribute direct revocation, scalar multiplication

## Abstract

Ciphertext-Policy Attribute-Based Encryption (CP-ABE) technology provides a new solution to address the security and fine-grained access control of traffic information in vehicular ad hoc networks (VANETs). However, in most CP-ABE schemes for VANETs, attribute revocation suffers from high system consumption and complex revocation operations, as well as from high computational overhead and low efficiency due to the use of bilinear pairwise operations. Based on this, this paper proposes a lightweight CP-ABE scheme that supports direct attribute revocation in VANETs. The scheme implements an agent-based direct attribute revocation mechanism by separating dynamic and static attributes of vehicle terminals, which reduces system consumption and simplifies the revocation operation process. The scheme uses scalar multiplication on elliptic curves instead of bilinear pairing operations and uses computational outsourcing techniques to reduce the terminal decryption cost and improve the efficiency of the scheme. The security and performance analysis shows that the overall efficiency of our scheme is better than the existing schemes under the premise of ensuring data confidentiality and integrity.

## 1. Introduction

A vehicular ad hoc network (VANET) [1] is a vast interactive network that carries important traffic information such as vehicle location, speed and route. It usually consists of an on-board unit (OBU) installed in the vehicle and a roadside unit (RSU) widely deployed at the roadside, and it aims to provide a comprehensive service platform for various applications. The widespread deployment of VANETs largely depends on a secure and reliable mechanism to provide effective data services in the transport system. Among many security issues, ensuring data integrity and confidentiality is the most important [2].

To ensure the confidentiality of data transmission in VANETs and to prevent data leakage and tampering, it is necessary to establish an effective access control scheme to ensure that data can only be accessed by authorized personnel. Compared to role-based access control [3,4], Ciphertext-Policy Attribute-Based Encryption (CP-ABE) [5,6] can provide more flexible and dynamic fine-grained access control. In 2005, Sahai and Waters [7] first proposed the concept of fuzzy identity-based encryption using bilinear pairing knowledge, and then further extended the concept of attribute-based encryption (ABE). An identity is considered as a set of descriptive attributes. ABE schemes are mainly divided into two categories: Key Policy Attribute-Based Encryption (KP-ABE) schemes and CP-ABE schemes. In 2006, Goyal et al. [8] proposed the first practical KP-ABE scheme, wherein the ciphertext is associated with a set of attributes and the user’s decryption key is associated with a monotonic tree access structure. In 2007, Bethencour et al. [9] introduced the tree access structure into the ciphertext and proposed the first CP-ABE scheme, wherein the user’s decryption key is associated with the attribute and the ciphertext is associated with the tree access structure. Subsequently, researchers conducted research on the revocability [10,11], computational outsourcing [12,13], multi-authority [14] and traceability [15] of the CP-ABE scheme, so the CP-ABE technology has become an important research direction to solve the access control of storage ciphertext. However, due to the use of bilinear pairing operations in most CP-ABE schemes, the overall efficiency of the scheme is reduced, which severely limits its use in IoT terminals with limited computational resources. Odelu et al. [16] and Ding et al. [17] proposed a CP-ABE scheme based on Elliptic Curve Cryptography (ECC). Compared to the bilinear pairing operation, the simple scalar multiplication over the elliptic curve used in the scheme has the advantages of lower computational overhead and higher efficiency.

In order to identify how to apply CP-ABE technology to VANETs to ensure the security of traffic information, researchers have proposed many schemes. Huang and Verma introduced CP-ABE technology to VANET, and proposed the first CP-ABE-based security policy implementation scheme in VANETs in [18], wherein different road conditions are considered as attributes, and the transmitted data are encrypted and protected in combination with a data access control strategy, but the effect of user and attribute revocation on the system is not considered in this scheme. Horng et al. [19] proposed an effective data access control CP-ABE scheme, wherein user and attribute revocation is provided by timestamp attributes, and cloud computing nodes are used to share the computational load of encryption and decryption. However, this scheme needs to re-encrypt the ciphertext in the process of user and attribute revocation and does not verify the data integrity in the process of outsourcing decryption. Aiming at the problem of limited computational resources of the vehicle terminal, Xia et al. [20] proposed a CP-ABE delegation scheme that allows the RSU to perform most of the computations to improve the decryption efficiency of the vehicle. Similarly, the scheme did not consider the impact of user and attribute revocation on the system, and the data integrity was not verified during the delegation decryption process. In order to adapt to the highly dynamic environment of VANETs and solve the data leakage and damage caused by outsourced data, Zhang et al. [21] proposed the concept of revocation with auditable users based on the CP-ABE algorithm, and used online/offline and verifiable outsourcing technology to improve the efficiency and ensure the correctness of the decryption. However, in the process of user revocation, the ciphertext and private keys of all non-revoked users need to be updated. Wang et al. [22] proposed a dynamic fine-grained access control scheme based on attribute encryption to solve this problem. However, the length of the ciphertext in this scheme is proportional to the number of authorized users, and the ciphertext must be updated when some authorized users are revoked or added.

However, the CP-ABE schemes in the aforementioned VANETs suffer from two problems. First, in terms of attribute revocation, most schemes implement attribute revocation by re-encrypting the ciphertext and updating the private keys of all unrevoked users, which is indirect revocation [23,24]. However, due to the high-speed mobility of vehicle terminals, dynamic attributes such as city, street and direction of travel are frequently updated and revoked, so using the indirect revocation mechanism will greatly increase the consumption of the system. Second, because the scheme uses bilinear pairing operations, it increases the computational overhead and reduces the overall efficiency, which is not suitable for use in vehicle terminals with limited computational resources. To solve the above two problems, this paper proposes a lightweight CP-ABE scheme that supports direct attribute revocation in VANETs. The main work is as follows:

(1) Aiming at the problem that using an indirect revocation mechanism to realize attribute revocation leads to large system consumption and complicated operation, by separating the static and dynamic attributes of the vehicle terminal, our scheme establishes a two-level decryption architecture of RSU and OBU and realizes the direct revocation of attributes based on an RSU agent, reducing system consumption due to frequent updating and revoking attributes.
(2) To address the problem of excessive computational overhead due to the use of bilinear pairing operations, our scheme is based on elliptic curve cryptography, using scalar multiplication instead of complex bilinear pairing operations, and outsourcing the decryption operations originally belonging to OBU to RSU to reduce computational costs and improve overall efficiency.
(3) The security analysis proves that our scheme is secure under a chosen plaintext attack. Theoretical and simulation experiments prove that our scheme is more efficient and less computationally expensive than the existing schemes.

The remainder of this paper is organized as follows: Section 2 briefly introduces the relevant knowledge covered in this paper. Section 3 presents the system model of our scheme and the specific implementation of the algorithm. Section 4 presents the security analysis of the scheme. Section 5 presents the performance analysis of our scheme. Finally, Section 6 concludes this work.

## 2. Preliminaries

### 2.1. Elliptic Curve Discrete Logarithm Problem

Elliptic curve cryptography is a public key cryptosystem based on the difficulty of solving the Elliptic Curve Discrete Logarithm Problem (ECDLP). The ECDLP problem is described as follows:

Given two points $P,G\in G_{E}$, where G is the generator elliptic curve group $G_{E}$, with order q, $k\in Z_{q}^{*}$ cannot be obtained within a polynomial-time algorithm such that P=kG.

### 2.2. Access Structure

Let $P=\{P_{1},\cdots ,P_{n}\}$ denote the set of participants such that $2^{P}=\{A|A\subseteq \{P_{1},\cdots ,P_{n}\}\}$. The set $A\subseteq 2^{P}$ is monotone if and only if for any subset B,C⊆P, C∈A if B∈A and B⊆C. A is said to be an access structure if A is a non-empty subset of $P=\{P_{1},\cdots ,P_{n}\}$, i.e.,$A\subseteq 2^{\left\{P_{1},\cdots ,P_{n}\right\}}\backslash \{\emptyset \}$. For any set D, D is an authorized set if D∈A, otherwise it is a non-authorized set.

### 2.3. Linear Secret Sharing Scheme

Suppose the set of participants is $P=\{P_{1},\cdots ,P_{n}\}$; if ∏ satisfies the following conditions, then ∏ is a linear secret sharing scheme (LSSS) defined on P.

(1) The secret shares held by each participant form a vector over $Z_{p}$.
(2) Each ∏ corresponds to a generator matrix M(l×n), and ρ:{1,2,⋯,l}→P maps each row (i=1,2,⋯,l) of M to a participant ρ(i), where ρ is an injective function. Consider the vector $v=(s,y_{2},\cdots ,y_{n})$, where $s\in Z_{p}$ is the secret value and $y_{2},\cdots ,y_{n}\in Z_{p}^{*}$ are randomly chosen, the l shares of the secret value s can be recorded as Mv, where $\lambda_{i}={\left(Mv\right)}_{i}$ is the *i*-th share of the secret value s, and belongs to ρ(i).

For any authorized set S of the access structure A, i.e., S∈A, define I={i:ρ(i)∈S}. Then, there exists a polynomial-time algorithm that computes a coefficient ${\left\{w_{i}\in Z_{p}\right\}}_{i\in I}$ such that $\sum_{i\in I}w_{i}M_{i}=(1,0,\cdots ,0)$ based on the matrix M. Thus, the secret value $s=\sum_{i\in I}w_{i}M_{i}\cdot v=\sum_{i\in I}w_{i}\lambda_{i}$ can be obtained. For non-authorized sets, the above coefficient does not exist and the secret value s cannot be obtained.

### 2.4. Decisional Diffie–Hellman Assumption

The definition of the decisional Diffie–Hellman (DDH) assumption on the elliptic curve is as follows:

Suppose $G_{q}$ is a cyclic group with a large prime number q as the order and G as the generator, a,b,c are three random numbers selected from $Z_{p}$. if the tuple R=(G,aG,bG,abG) and D=(G,aG,bG,cG) are computationally indistinguishable, then it is called the *DDH* assumption. Attacker A has advantage ε in distinguishing the *DDH* assumption of tuple R and D, if

(1) $$Adv_{A}^{DDH}(\kappa )=|\mathrm{Pr}[A(R)=1]-\mathrm{Pr}[A(D)=1]|\geq \varepsilon$$

**Definition 1.**  *The DDH assumption holds if there is no polynomial-time algorithm to solve the DDH problem with non-negligible advantage*.

## 3. Proposed Scheme

### 3.1. System Mode

**Figure 1:**
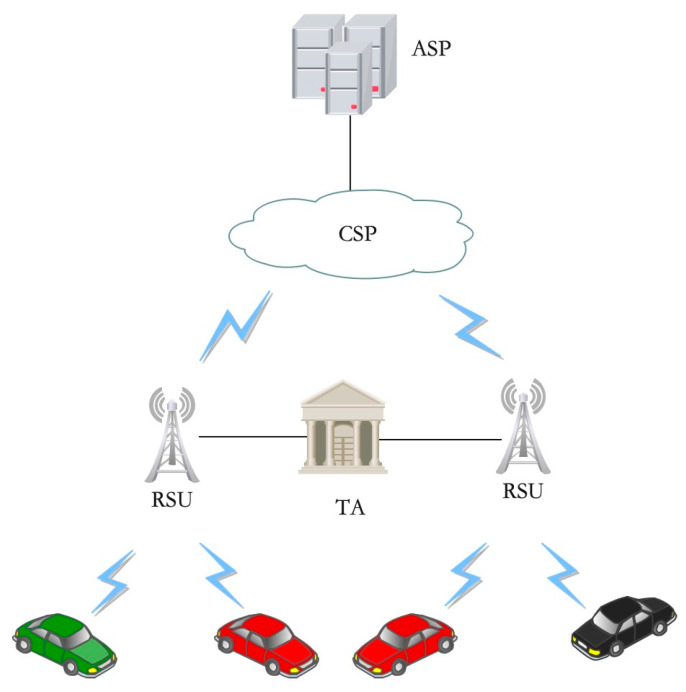
System model.

In order to provide an efficient attribute revocation mechanism for VANETs, we propose a lightweight CP-ABE scheme with direct attribute revocation. The system model consists of five types of different entities: the Trust Authority (TA), the Cloud Service Providers (CSPs), the Application Service Providers (ASPs), the Roadside Units (RSUs), and the Onboard Units (OBUs), as shown in Figure 1.

(1) TA: The TA is a fully trusted server with high computing power, regulated by government authorities and always online. The TA is responsible for initializing system parameters and generating system public and master keys. The TA generates attribute keys for all attributes defined by the system and publishes their public keys. The attributes defined by the system are divided into static and dynamic attributes. Among them, static attributes include vehicle type, make, registration number and company, etc., which will not change in a short time for the OBU; dynamic attributes include the current driving city, street and driving direction, etc., which will change frequently for the OBU. The TA is responsible for RSU and OBU registration, binds the unique user identity identifier UID_RSU or UID_OBU for the user, and generates the user decryption key according to its attributes. In addition, the TA will also generate a certificate for the OBU to authenticate with the RSU.
(2) CSPs: The CSPs have abundant storage space, store encrypted data uploaded by ASPs or OBUs and send encrypted data to authorized entities according to the request. In the scheme design of this paper, the CSP is honest but curious, i.e., it will honestly perform related tasks and additionally infer private information.
(3) ASPs: The ASPs are responsible for providing applications or services to vehicles, such as GPS service providers who can collect traffic data provided by vehicles from CSPs and then process the collected data to serve different users through different GPS services. Alternatively, if a taxi company only wants to provide services to its vehicles in a certain area, it can encrypt the application data according to its own defined access policy and upload it to the CSP.
(4) RSUs: The RSUs are widely deployed at roadsides and intersections, have relatively abundant computing and storage space and are regulated by government departments. When the local traffic management department deploys the RSU, it will apply to the TA for the unique identity UID_RSU and the attribute decryption key according to the attributes such as the deployed city, road and lane direction.
(5) OBUs: When the vehicle production is completed, the company will apply to the TA for the unique identity UID_OBU, attribute decryption key and digital certificate for OBU through the local traffic management department according to its vehicle type, brand and registration number.

### 3.2. Specific Implementation

**Figure 2:**
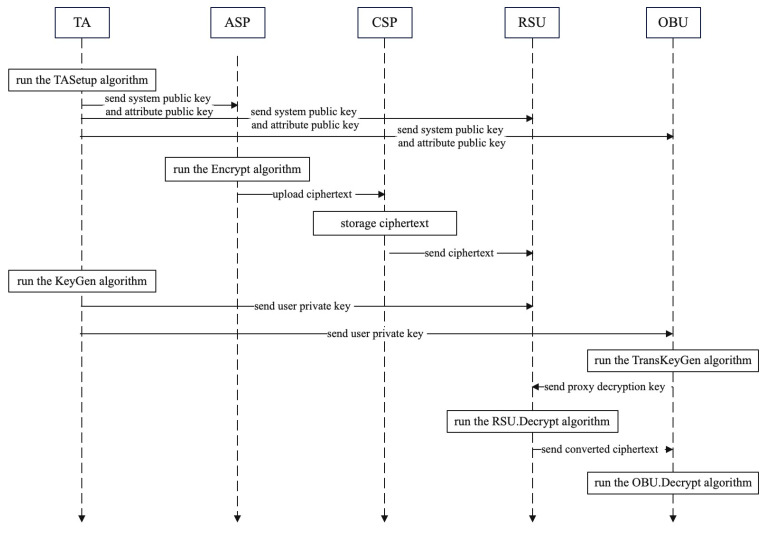
System flowchart.

To reduce system consumption and simplify revocation operations, we establish a two-stage decryption architecture and use an RSU proxy to implement attribute direct revocation. To reduce the computational overhead, we use scalar multiplication based on elliptic curves for encryption and decryption computations. In addition, to further reduce the consumption of computational resources of the OBU, we outsource the decryption operation originally belonging to the OBU to the RSU and increase the verification of data integrity. Specifically, our proposed lightweight CP-ABE scheme supporting direct attribute revocation in VANETs consists of the following six algorithms: Setup, TASetup, KeyGen, TransKeyGen, Encrypt and Decrypt. The system flowchart is shown in Figure 2. A detailed description of the above algorithms is given below:

#### 3.2.1. Setup

Setup(λ)→params: This algorithm takes the security parameter λ as input and outputs the system public parameter params={GF(q),E,G,U,H}, where GF(q) represents a finite field with prime number q as the order, E represents an elliptic curve selected on the finite field, G is a base point selected on the elliptic curve E with p as the order, $U=\{a_{1},a_{2},\cdots ,a_{m}\}$ represents a set of attributes and $H:\{0,1\}\to Z_{p}^{*}$ is a hash function selected by the system to map the user identity UID to the elements in $Z_{p}^{*}$.

#### 3.2.2. TASetup

TASetup(params,U)→(PK,MSK,ASK,APK): This algorithm takes the system public parameter params and the system attribute set U as input and takes the system public key PK, the system master private key MSK, the attribute private key ASK and the attribute public key APK as output. The TA randomly selects element n from $Z_{p}^{*}$ as the master private key and calculates nG as the public key, namely PK=nG, MSK=n. For each attribute $a_{i}\in U$ defined in the system, the TA will randomly select $k_{i}\in Z_{p}^{*}$ as the attribute private key and use $k_{i}G$ as the attribute public key $PK_{a_{i}}$, namely $ASK=\{k_{i}\}$, $APK=\{PK_{a_{i}}\}$.

#### 3.2.3. KeyGen

$KeyGen(params,MSK,S,UID)\to SK_{i,UID}$: This algorithm is run by the TA, takes the system public parameter params, the system master private key MSK, the user attribute set S and the user unique identity UID as input and outputs the user private key $SK_{i,UID}$ associated with user identity and attributes. In order to facilitate the distinction, this paper records the user private key applied for by RSU as $SK_{i,UID\_RSU}$, and the user private key applied by OBU as $SK_{i,UID\_OBU}$. In addition, when OBU applies to the TA for the attribute decryption key, the TA will also generate the digital authentication certificate $Cert_{OBU}$ according to the static attribute set S owned by the OBU, attribute validity period and identity, etc., for access authentication with RSU. When the TA generates the corresponding attribute decryption private key for the user, it will bind the attribute private key $k_{i}$ of the attribute $a_{i}$ possessed by the user with the user identity UID, namely $SK_{i,UID}=k_{i}+H(UID)n$. When the OBU applies for the attribute decryption key from the TA, the TA will update the digital authentication certificate $Cert_{OBU}$, adding a new static attribute owned by the OBU and the validity period of the attribute to it.

#### 3.2.4. TransKeyGen

$TransKeyGen(params,SK_{i,UID\_OBU})\to (AK_{i,UID\_OBU},TK)$: The algorithm is run by OBU, takes the system public parameters params and the decryption key $SK_{i,UID\_OBU}$ obtained from TA as input and outputs the proxy decryption key $AK_{i,UID\_OBU}$ and the converted key TK. When the OBU receives the relevant attribute decryption key, it will randomly select an element t from $Z_{p}^{*}$ to calculate the proxy decryption key and converted key, namely $AK_{i,UID\_OBU}=SK_{i,UID\_OBU}-t$, TK=t.

#### 3.2.5. Encrypt

$Encrypt(params,M,(A_{s},\rho_{s}),(A_{d},\rho_{d}))\to CT$: The algorithm is run by the data owner ASP or OBU, takes the system public parameter params, message M and static and dynamic attribute access control structure $\left(A_{s},\rho_{s}\right)$, $\left(A_{d},\rho_{d}\right)$ as input, and outputs ciphertext CT. The data owner creates the static attribute LSSS access structure $\left(A_{s},\rho_{s}\right)$ and the dynamic attribute LSSS access structure $\left(A_{d},\rho_{d}\right)$ according to the defined access control strategy, where $A_{s}$ and $A_{d}$ represent the access control matrix of $l_{s}\times m_{s}$ and $l_{d}\times m_{d}$, respectively, and $\rho_{s}(x)$ and $\rho_{d}(x)$ represent each row in the access matrix the corresponding attributes. Next, the data owner randomly selects two elements $s,d\in Z_{p}^{*}$ for static and dynamic attribute encryption, respectively, where $s_{x}$ and $d_{x}$ in $sG=(s_{x},s_{y})$ and $dG=(d_{x},d_{y})$ are, respectively, used as symmetric keys to perform symmetric encryption and decryption of data, while $s_{y}$ and $d_{y}$ are used for data integrity verification. The specific process of data encryption is as follows:

(1) Static attribute encryption: First, the data owner uses $s_{x}$ as a symmetric key to encrypt data M, that is, $C_{M_{s}}=Enc(M,s_{x})$, and uses $s_{y}$ as a key to obtain the message authentication code of data M, that is, $MAC_{M_{s}}=HMAC(M,s_{y})$. Then, it randomly selects two vectors $v_{s},u_{s}\in Z_{p}^{m_{s}}$, where the first element of $v_{s}$ is s, and the first element of $u_{s}$ is 1, and calculates $\lambda_{x_{s}}=A_{x_{s}}\cdot v_{s}$ and $\omega_{x_{s}}=A_{x_{s}}\cdot u_{s}$, respectively, where $A_{x_{s}}$ represents the x-th row of the matrix $A_{s}$. Next, it calculates $C_{x_{s},1}=\lambda_{x_{s}}G+\omega_{x_{s}}PK_{\rho_{s}(x)}$, $C_{x_{s},2}=\omega_{x_{s}}G$. Finally, the ciphertext encrypted by the static attribute is computed as

(2) $$CT_{s}=\{(A_{s},\rho_{s}),C_{M_{s}},MAC_{M_{s}},\forall x_{s}\in [0,l_{s}-1]:C_{x_{s},1},C_{x_{s},2}\}$$

(2) Dynamic attribute encryption: Similar to the static attribute encryption process. First, the data owner uses $d_{x}$ as a symmetric key to encrypt data $C_{M_{s}}$, that is, $C_{M_{s\_d}}=Enc(C_{M_{s}},d_{x})$, and uses $d_{y}$ as a key to obtain the message authentication code of data $C_{M_{s}}$, that is, $MAC_{M_{s\_d}}=HMAC(C_{M_{s}},d_{y})$. Then, it randomly selects two vectors $v_{d},u_{d}\in Z_{p}^{m_{d}}$, where the first element of $v_{d}$ is d, and the difference from static attribute encryption is that the first element of $u_{d}$ is 0, and calculates $\lambda_{x_{d}}=A_{x_{d}}\cdot v_{d}$ and $\omega_{x_{d}}=A_{x_{d}}\cdot u_{d}$, respectively, where $A_{x_{d}}$ represents the x-th row of the matrix $A_{d}$. Next, it calculates $C_{x_{d},1}=\lambda_{x_{d}}G+\omega_{x_{d}}PK_{\rho_{d}(x)}$, $C_{x_{d},2}=\omega_{x_{d}}G$. Finally, the ciphertext encrypted by the dynamic attribute is computed as

(3) $$CT_{d}=\{(A_{d},\rho_{d}),C_{M_{s\_d}},MAC_{M_{s\_d}},\forall x_{d}\in [0,l_{d}-1]:C_{x_{d},1},C_{x_{d},2}\}$$

After encryption of static attributes and dynamic attributes, the encrypted ciphertext of data M is finally computed as

(4) $$CT=\{CT_{s}\backslash C_{M_{s}},CT_{d}\}$$

#### 3.2.6. Decrypt

When the OBU requests to access data, it will send the digital certificate $Cert_{OBU}$ to the RSU for identity authentication. After the RSU obtains $Cert_{OBU}$, it will judge whether the identity of the OBU is valid and obtain the valid static attribute set owned by the OBU according to the user attributes and attribute validity period contained in $Cert_{OBU}$. After the identity authentication is passed, the RSU will submit the corresponding data access request to the CSP. After the RSU receives the ciphertext sent by the CSP, it will judge whether it meets the access policy preset by the data owner according to its own dynamic attribute set and the obtained the OBU static attribute set, and then decrypt the ciphertext if it is satisfied. The specific decryption process is as follows:

The data decryption consists of two parts, namely the data outsourcing decryption algorithm RSU.Decrypt run by RSU and the data local decryption algorithm OBU.Decrypt run by OBU.

(1) $RSU.Decrypt(params,CT,SK_{i,UID\_RSU},AK_{i,UID\_OBU})\to CT^{\prime }$: The algorithm takes the system public parameter params, the ciphertext CT, the user private key $SK_{i,UID\_RSU}$ of RSU and the proxy decryption key $AK_{i,UID\_OBU}$ provided by OBU as input, and outputs the converted ciphertext $CT^{\prime }$. The algorithm consists of two stages.

(a) First, the RSU uses its own key $SK_{i,UID\_RSU}$ to decrypt the ciphertext CT encrypted by the dynamic attribute access control structure and verify the integrity of the data. Using $SK_{i,UID\_RSU}$, $C_{x_{d},1}$ and $C_{x_{d},2}$ to calculate, RSU can be obtained as

(5) $$\begin{array}{l} C_{x_{d}}=C_{x_{d},1}-C_{x_{d},2}SK_{i,UID\_RSU}\\ =\lambda_{x_{d}}G+\omega_{x_{d}}PK_{\rho_{d}(x)}-\omega_{x_{d}}G(k_{\rho_{d}(x)}+H(UID\_RSU)n)\\ =\lambda_{x_{d}}G+\omega_{x_{d}}k_{\rho_{d}(x)}G-\omega_{x_{d}}k_{\rho_{d}(x)}G-\omega_{x_{d}}H(UID\_RSU)nG\\ =\lambda_{x_{d}}G-\omega_{x_{d}}H(UID\_RSU)nG \end{array}$$

(6) $$\begin{array}{l} \sum_{\rho_{d}(x)\in S}c_{x_{d}}C_{x_{d}}\\ =\sum_{\rho_{d}(x)\in S}c_{x_{d}}(\lambda_{x_{d}}G-\omega_{x_{d}}H(UID\_RSU)nG)\\ =\sum_{\rho_{d}(x)\in S}c_{x_{d}}(A_{x_{d}}v_{d}G-A_{x_{d}}u_{d}H(UID\_RSU)nG)\\ =\sum_{\rho_{d}(x)\in S}c_{x_{d}}A_{x_{d}}v_{d}G-\sum_{\rho_{d}(x)\in S}c_{x_{d}}A_{x_{d}}u_{d}H(UID\_RSU)nG\\ =dG \end{array}$$

where since the first element of $v_{d}$ is d and the first element of $u_{d}$ is 0, $\sum_{\rho_{d}(x)\in S}c_{x_{d}}A_{x_{d}}v_{d}=d$ and $\sum_{\rho_{d}(x)\in S}c_{x_{d}}A_{x_{d}}u_{d}=0$.

After obtaining $dG=(d_{x},d_{y})$, the symmetric key $d_{x}$ and the key d for data integrity verification can be obtained. RSU uses $d_{x}$ to perform symmetric decryption can obtain the data $C_{M_{s}}$, and uses the key $d_{y}$ to calculate whether $HMAC(C_{M_{s}},d_{y})$ is equal to $MAC_{M_{s\_d}}$ contained in the ciphertext CT to judge whether the data integrity of the ciphertext is maliciously damaged during data transmission and storage.

(b) The second stage is that RSU obtains the decryption key of relevant attributes from the proxy decryption key $AK_{i,UID\_OBU}$ provided by OBU according to the obtained OBU effective static attribute set, and then decrypts the part encrypted by the static attribute access control structure in the ciphertext CT, obtains the converted ciphertext $CT^{\prime }$ and sends it to the OBU. Using $AK_{i,UID\_OBU}$, $C_{x_{s},1}$ and $C_{x_{s},2}$ to calculate, RSU can be obtained as:

(7) $$\begin{array}{l} C_{x_{s}}=C_{x_{s},1}-C_{x_{s},2}AK_{i,UID\_OBU}\\ =\lambda_{x_{s}}G+\omega_{x_{s}}PK_{\rho_{s}(x)}-\omega_{x_{s}}G(k_{\rho_{s}(x)}+H(UID\_OBU)n-t)\\ =\lambda_{x_{s}}G+\omega_{x_{s}}k_{\rho_{s}(x)}G-\omega_{x_{s}}k_{\rho_{s}(x)}G-\omega_{x_{s}}H(UID\_OBU)nG-t\omega_{x_{s}}G\\ =\lambda_{x_{s}}G-\omega_{x_{s}}H(UID\_OBU)nG-t\omega_{x_{s}}G \end{array}$$

(8) $$\begin{array}{l} C=\sum_{\rho_{s}(x)\in S}c_{x_{s}}C_{x_{s}}\\ =\sum_{\rho_{s}(x)\in S}c_{x_{s}}(\lambda_{x_{s}}G-\omega_{x_{s}}H(UID\_OBU)nG-t\omega_{x_{s}}G)\\ =\sum_{\rho_{s}(x)\in S}c_{x_{s}}(A_{x_{s}}v_{s}G-A_{x_{s}}u_{s}H(UID\_OBU)nG-tA_{x_{s}}u_{s}G)\\ =\sum_{\rho_{s}(x)\in S}c_{x_{s}}A_{x_{s}}v_{s}G-\sum_{\rho_{s}(x)\in S}c_{x_{s}}A_{x_{s}}u_{s}H(UID\_OBU)nG-\sum_{\rho_{s}(x)\in S}c_{x_{s}}A_{x_{s}}u_{s}tG\\ =sG-H(UID\_OBU)nG-tG \end{array}$$

where since the first element of $v_{s}$ is s and the first element of $u_{s}$ is 1, $\sum_{\rho_{s}(x)\in S}c_{x_{s}}A_{x_{s}}v_{s}=s$ and $\sum_{\rho_{s}(x)\in S}c_{x_{s}}A_{x_{s}}u_{s}=1$.

Finally, the converted ciphertext obtained by RSU is $CT^{\prime }=\{C_{M_{s}},MAC_{M_{s}},C\}$.

(2) $OBU.Decrypt(params,CT^{\prime },TK)\to M$: The algorithm takes the system public parameter params, the converted ciphertext $CT^{\prime }$ and the converted key TK as input, and outputs the original data information M. After the OBU obtains $CT^{\prime }$, it can be calculated by using PK and TK as
  (9) $$\begin{array}{l} C+H(UID\_OBU)nG+tG\\ =(sG-H(UID\_OBU)nG-tG)+H(UID\_OBU)nG+tG\\ =sG \end{array}$$

After obtaining $sG=(s_{x},s_{y})$, the symmetric key $s_{x}$ and the key $s_{y}$ for data integrity verification can be obtained. OBU uses the symmetric key $s_{x}$ to perform symmetric decryption can obtain the data M and uses the key $s_{y}$ to calculate whether $HMAC(M,s_{y})$ is equal to $MAC_{M_{s}}$. If they are equal, it means that the data M obtained by OBU decryption has not been maliciously tampered with.

#### 3.2.7. Direct Revocation

(1) User revocation: In the scheme proposed in this paper, for the user revocation of an OBU, the local traffic management department can initiate a user revocation request to the TA, and the TA will add the certificate $Cert_{OBU}$ of the OBU user to the certificate revocation list (CRL) to make the RSU reject the access authentication of the OBU user.
(2) Dynamic attribute revocation: Since the part of the ciphertext encrypted with the dynamic attribute access control policy is decrypted by the RSU, when the OBU leaves the coverage area of a particular RSU, it no longer receives the converted ciphertext sent by the RSU, thus realizing the direct revocation of the dynamic attributes of the vehicle terminal.
(3) Static attribute revocation: In the design of this scheme, when the OBU requests an attribute decryption key for an attribute, the TA sets the validity period of the attribute in the certificate $Cert_{OBU}$. Therefore, after the OBU sends the certificate $Cert_{OBU}$ to the RSU, the RSU can obtain the valid static attribute set of the OBU and then obtain the decryption key of the valid attribute from the proxy decryption key $AK_{i,UID\_OBU}$. If a static attribute has expired, the valid static attribute set obtained by the RSU will not contain that attribute. When a static attribute has not expired but still needs to be revoked, the local traffic management department can initiate an attribute revocation request to the TA, and the TA will modify the certificate $Cert_{OBU}$ of the OBU user and delete the attribute from $Cert_{OBU}$. The effective static attribute set obtained by the RSU will also not contain this attribute, thereby realizing the direct revocation of the static attribute.

## 4. Security Discussion and Analysis

### 4.1. Security Discussion

The scheme proposed in this paper has anti-collusion security, forward security and correctness of outsourced decryption.

(1) Anti-collusion security: In the scheme proposed in this paper, the user keys distributed from TA to OBU are all bound to their unique identities. Therefore, even if multiple users who do not meet the access structure collude with each other to share keys, due to their different identities, it is impossible to eliminate redundant elements by combination to obtain the hidden secret value, thus ensuring that the scheme has anti-collusion security.
(2) Forward security: For a given user revocation, the TA adds the certificate of the OBU to the CRL so that the OBU cannot be connected to the RSU and the decryption of the ciphertext cannot be completed by the RSU. For a particular attribute, the direct revocation of the attribute can be realized based on the RSU proxy. The above two revocation methods ensure the forward security of the proposed scheme.
(3) Correctness of outsourced decryption: In the scheme proposed in this paper, OBU can calculate the keys $s_{x}$ and $s_{y}$ after obtaining sG, and then use the key $s_{x}$ to obtain the data M, use the key $s_{y}$ to calculate $HMAC(M,s_{y})$ and compare it with the $MAC_{M_{s}}$ contained in the converted ciphertext to judge the correctness of the outsourced decryption.

### 4.2. Security Model

The security model of the scheme proposed in this paper is defined based on the game between the challenger and the attacker, specifically described as

(1) Initialization: The TA first runs the Setup and TASetup algorithms to generate the system public parameters params, public key PK and attribute public key APK to provide to the attacker. The attacker then selects a set of challenge access structures $\left\{(A_{s},\rho_{s}),(A_{d},\rho_{d})\right\}$ to send to the challenger.
(2) Phase 1: The attacker can request to query the private key of any attribute not in the challenge access structure.
(3) Challenge: The attacker submits two randomly selected messages $M_{0}$ and $M_{1}$ of equal length to the challenger. The challenger first randomly selects β∈{0,1} and then encrypts the message $M_{\beta }$ according to the challenge access structure $\left\{(A_{s},\rho_{s}),(A_{d},\rho_{d})\right\}$ submitted by the attacker.
(4) Phase 2: As in Phase 1, the attacker can request to query any attribute private key that cannot be used to decrypt the challenge ciphertext.
(5) Guess: The attacker outputs the guess result $\beta^{\prime }$ of β. The advantage of the attacker in this game process is defined as Pr[β′=β]−12.

**Definition 2.**  *If any polynomial time attacker cannot win with a non-negligible advantage in the game process, the scheme proposed in this paper is indistinguishable under chosen plaintext attack, which is called IND-CPA security.*

### 4.3. Security Analysis

**Theorem 1.**  *If the DDH assumption under elliptic curves holds, an attacker who does not have polynomial time can break the scheme in this paper with a non-negligible advantage.*

**Proof.**  Suppose there is a polynomial time attacker A who can break the scheme in this paper with a non-negligible advantage ε>0 under the security model defined in this paper, then challenger B can solve the DDH problem with a ε2 advantage. The proof process is as follows:

Let $G_{p}$ be a cyclic group with a large prime number p as the order and a base point G on the elliptic curve E as the generator. Challenger B selects two random numbers a,b from $Z_{p}$, randomly selects an element R from $G_{p}$, and randomly selects β∈{0,1}. If  β=0, challenger B makes the tuple (G,aG,bG,Z)=(G,aG,bG,abG); otherwise, let the tuple (G,aG,bG,Z)=(G,aG,bG,R). Finally, challenger B sends the tuple (G,aG,bG,Z) to simulator C. Simulator C will replace challenger B to interact with attacker A.

(1) Initialization: Simulator C first runs the Setup and TASetup algorithms to generate system public parameters params, master private key MSK=n, system public key PK=nG, attribute private key $k_{i}$ and attribute public key $PK_{a_{i}}=k_{i}G$ for each attribute $a_{i}$. Then, simulator C provides params, PK and $PK_{a_{i}}$ to attacker A. Finally, simulator C initializes a list H for recording interactions with attacker A. Attacker A chooses a set of challenge access structures $\left\{(A_{s},\rho_{s}),(A_{d},\rho_{d})\right\}$ and sends them to simulator C.
(2) Phase 1: Attacker A can submit $\left(a_{i},UID\right)$ to simulator C to query the private key of any attribute not in the challenge access structure. The simulator C verifies whether the element $\left((a_{i},UID),SK_{i,UID}\right)$ is already contained in the list H. If $\left(a_{i},UID\right)$ already exists in the list H, the simulator C responds with the $SK_{i,UID}$ in $\left((a_{i},UID),SK_{i,UID}\right)$. Otherwise, the simulator C randomly selects $h\in Z_{p}^{*}$, calculates $SK_{i,UID}=k_{i}a+h$ as a response and stores the element $\left((a_{i},UID),SK_{i,UID}\right)$ in the list H.
(3) Challenge: Attacker A submits two randomly selected messages $M_{0}$ and $M_{1}$ of equal length to Simulator C. Simulator C first randomly selects β∈{0,1}. Then, Simulator C randomly selects two elements $s,d\in Z_{p}^{*}$, uses $s_{x}$ as a symmetric key to encrypt data $M_{\beta }$ to obtain $C_{M_{s}}$, uses $s_{y}$ as a key to obtain the message authentication code $MAC_{M_{s}}$ of data $M_{\beta }$, uses $d_{x}$ as a symmetric key to encrypt data $C_{M_{s}}$ to obtain $C_{M_{s\_d}}$ and uses $d_{y}$ as a key to obtain the message authentication code $MAC_{M_{s\_d}}$ of data $C_{M_{s}}$. Next, simulator C randomly selects four vectors $v_{s},u_{s}\in Z_{p}^{m_{s}}$ and $v_{d},u_{d}\in Z_{p}^{m_{d}}$, where the first element of $v_{s}$ is s, the first element of $u_{s}$ is 1, the first element of $v_{d}$ is d, and the first element of $u_{d}$ is 0, and calculates $\lambda_{x_{s}}=A_{x_{s}}\cdot v_{s}$, $\omega_{x_{s}}=A_{x_{s}}\cdot u_{s}$, $\lambda_{x_{d}}=A_{x_{d}}\cdot v_{d}$ and $\omega_{x_{d}}=A_{x_{d}}\cdot u_{d}$. Finally, the simulator C calculates and obtains $C_{x_{s},1}=\lambda_{x_{s}}G+\omega_{x_{s}}k_{\rho_{s}(x)}Z$, $C_{x_{s},2}=\omega_{x_{s}}bG$, $C_{x_{d},1}=\lambda_{x_{d}}G+\omega_{x_{d}}k_{\rho_{d}(x)}Z$ and $C_{x_{d},2}=\omega_{x_{d}}bG$. The simulator C generates the challenge ciphertext CT of the information $M_{\beta }$ and sends it to the attacker A.
  (10) $$\begin{array}{l} CT=\{(A_{s},\rho_{s}),(A_{d},\rho_{d}),C_{M_{s\_d}},MAC_{M_{s}},MAC_{M_{s\_d}},\\ \forall x_{s}\in [0,l_{s}-1]:C_{x_{s},1},C_{x_{s},2},\forall x_{d}\in [0,l_{d}-1]:C_{x_{d},1},C_{x_{d},2}\} \end{array}$$
(4) Phase 2: Similar to Phase 1.
(5) Guess: Attacker A outputs the guess result $\beta^{\prime }$ of β. If $\beta^{\prime }=\beta$, simulator C outputs 0 to indicate that the guess result is Z=abG. Otherwise, simulator C outputs 1 to indicate that the guess result is Z=R.

If Z=abG, then $C_{x_{s},1}-C_{x_{s},2}SK_{i,UID\_OBU}=\lambda_{x_{s}}G-hb\omega_{x_{s}}G$, $C_{x_{d},1}-C_{x_{d},2}SK_{i,UID\_RSU}=\lambda_{x_{d}}G-hb\omega_{x_{d}}G$, indicating that the challenge ciphertext CT is encrypted under the challenge access structure submitted by attacker A. Since the advantage of attacker A is ε, the probability that attacker A correctly guesses β in this case is

(11) Pr[C(G,aG,bG,Z=abG)=0]=12+ε

If Z=R, since R is randomly selected, the probability that attacker A correctly guesses β in this case is

(12) Pr[C(G,aG,bG,Z=R)=0]=12

In summary, the advantage of Simulator C is

(13) 12(Pr[C(G,aG,bG,Z=abG)=0]+Pr[C(G,aG,bG,Z=R)=0])−12=ε2

The above proof shows that the scheme proposed in this paper satisfies *IND-CPA* security under the *DDH* assumption. □

## 5. Performance Analysis

### 5.1. Theoretical Analysis

Table 1 shows the functional comparison of our scheme with other schemes. As can be seen from the table, the schemes in references [16,17] and our scheme use scalar multiplication on elliptic curves, while the schemes in references [18,20] are based on bilinear pairing for data encryption and decryption operations. Compared to the schemes in [16,17,18,20], our scheme use computational outsourcing techniques to reduce the computational burden of decryption for the user. Compared to the schemes in [16,17,18,20], our scheme adds data integrity verification to verify whether the ciphertext is maliciously corrupted during transmission, storage and computational outsourcing.

Table 2 shows the computational overhead of our scheme compared to other schemes in terms of user encryption, user decryption and outsourced decryption. The descriptors used in the table are as follows: $E_{c}$, $E_{g}$, $E_{T}$ and $E_{p}$ denote the computational overhead of scalar multiplication of elliptic curves, the computational overhead of exponential operations in bilinear group G, the computational overhead of exponential operations in $G_{T}$ and the computational overhead of bilinear pairwise operations, respectively. H is the computational overhead of the hash function. L is the number of attributes contained in the access control structure. M is the minimum number of attributes required to decrypt the ciphertext. N is the number of all attributes contained in the system. ω is the number of attributes in the AND gate structure. As can be seen from the table, compared to the schemes in [16,17,18,20], our scheme makes the computational overhead in the user decryption process stable by using computational outsourcing. Our scheme requires less computational overhead in the outsourcing process compared to the scheme in [20].

### 5.2. Experiment Analysis

**Figure 3:**
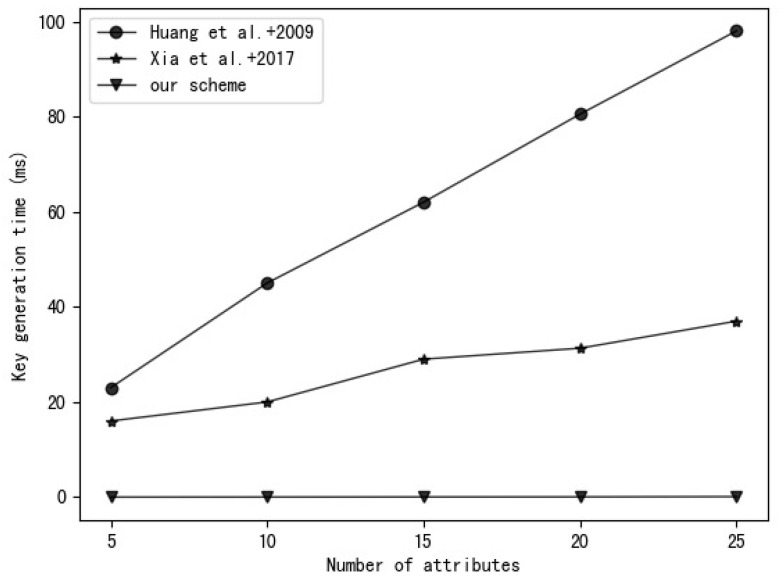
Comparison of Huang et al. [18], Xia et al. [20] and our scheme in terms of key generation time.

**Figure 4:**
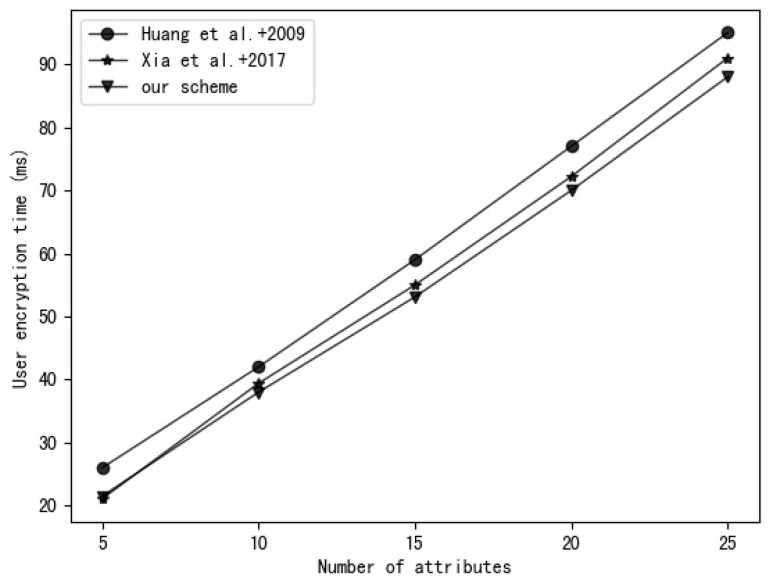
Comparison of Huang et al. [18], Xia et al. [20] and our scheme in terms of user encryption time.

**Figure 5:**
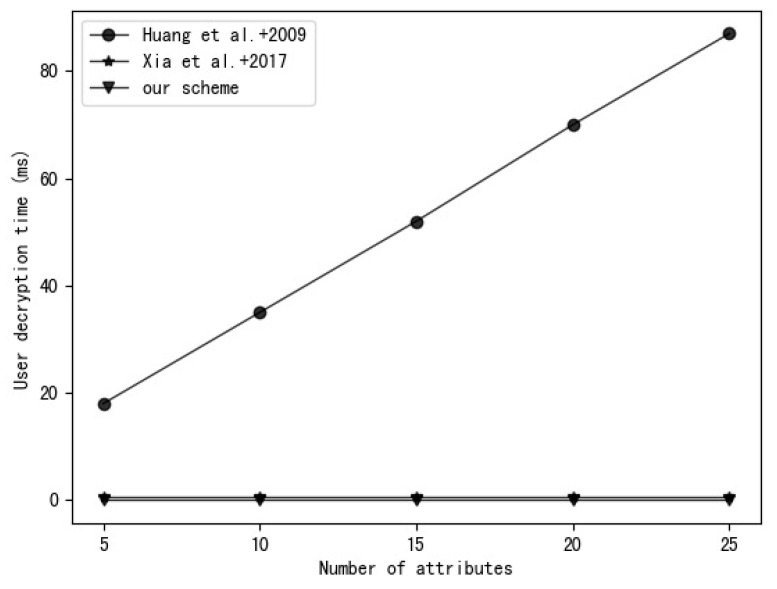
Comparison of Huang et al. [18], Xia et al. [20] and our scheme in terms of user decryption time.

**Figure 6:**
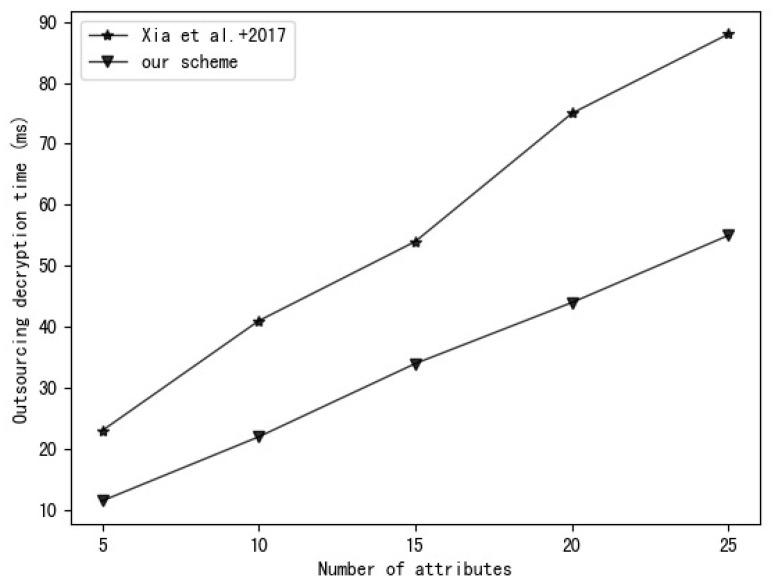
Comparison of Xia et al. [20] and our scheme in terms of outsourced decryption time.

Our experimental environment uses a 2.6 GHz Intel Core i7 processor, Ubuntu Linux 16.04.7 system. The experimental code is written based on the charm-crypto framework and python 3.7 and uses a 160-bit elliptic curve group in a supersingular curve $y^{2}=x^{3}+x$ based on a 512-bit finite field. A comparison of the time required to perform various operations in this environment is shown in Table 3. The experimental results are the average of 30 rounds of experiments. Figure 3, Figure 4, Figure 5 and Figure 6, respectively, show the calculation time comparison between our scheme and the schemes in [18] and [20] in the process of key generation, user encryption, user decryption and outsourced decryption.

It can be seen from Figure 3 that the key generation time in the schemes of [18] and [20] grows with the increase in user attributes, but the key generation time in our scheme is almost constant. It can be seen from Figure 4 that the user encryption time in the schemes of [18,20] and our scheme grows with the increase in attributes in the access control policy, but the encryption time in our scheme is relatively small.

From Figure 5, we can see that the data decryption time of the scheme in [18] increases with the number of attributes. However, the scheme in [20] and our scheme use computation outsourcing technology, so the data decryption time does not increase due to the complexity of the access policy. Additionally, compared to the scheme in [20], our scheme requires less decryption time and is more efficient. It can be seen from Figure 6 that the outsourced decryption time of the scheme in [20] and our scheme increases with the increase in the number of attributes, but the outsourced decryption time of our scheme is shorter than that of the scheme in [20], and with the increase in the number of attributes, the time difference between the two schemes gradually increases. This is because the scalar multiplication used in our scheme has the characteristics of low computational overhead and high efficiency compared to the bilinear pairing operation used in [20].

## 6. Conclusions

In this paper, we propose a lightweight CP-ABE scheme that supports direct attribute revocation. The scheme establishes a two-step decryption architecture for RSU and OBU by separating dynamic and static attributes of in-vehicle terminals and achieves efficient attribute revocation without re-encrypting the ciphertext and modifying the private key of unrevoked users to reduce system consumption. The scheme is based on elliptic curve cryptography and uses scalar multiplication to perform data computation, which improves the overall efficiency and reduces the computational overhead. A fixed ciphertext length can effectively reduce the communication resource consumption in the VANET environment, but in our scheme, the ciphertext length increases with the number of attributes in the access control policy. Therefore, in future work, we will further improve the scheme in terms of ciphertext length fixing.

## Figures and Tables

**Table 1:** Function comparison.

Scheme	Bilinear Pairing	Scalar Multiplication	Outsourced Computing	Integrit yVerification
Scheme in [16]	No	Yes	No	No
Scheme in [17]	No	Yes	No	No
Scheme in [18]	Yes	No	No	No
Scheme in [20]	Yes	No	Yes	No
Our Scheme	No	Yes	Yes	Yes

**Table 2:** Computational cost comparison.

Scheme	User Encryption	User Decryption	Outsourcing Decryption
Scheme in [16]	$(N-\omega +2)E_{c}$	$(N-\omega +3)E_{c}$	—
Scheme in [17]	$(3L+1)E_{c}$	$2ME_{c}$	—
Scheme in [18]	$(2L+1)E_{g}+E_{T}+H$	$(2M+1)E_{p}$	—
Scheme in [20]	$(2L+1)E_{g}+E_{T}$	$E_{p}$	$(2M+1)E_{p}+E_{T}$
Our Scheme	$3LE_{c}$	$2E_{c}$	$2ME_{c}$

**Table 3:** Execution time of operations.

Operations	$E_{p}$	$E_{g}$	$E_{c}$
Time	3.5 ms	1.76 ms	1.16 ms

## Data Availability

Not applicable.
